# Association of Metformin Use With Cancer-Specific Mortality in Hepatocellular Carcinoma After Curative Resection: A Nationwide Population-Based Study

**DOI:** 10.1097/MD.0000000000003527

**Published:** 2016-04-29

**Authors:** Young-Seok Seo, Yun-Jung Kim, Mi-Sook Kim, Kyung-Suk Suh, Sang Bum Kim, Chul Ju Han, Youn Joo Kim, Won Il Jang, Shin Hee Kang, Ha Jin Tchoe, Chan Mi Park, Ae Jung Jo, Hyo Jeong Kim, Jin A Choi, Hyung Jin Choi, Michael N. Polak, Min Jung Ko

**Affiliations:** From the Department of Radiation Oncology (Y-SS, M-SK, WIJ), Korea Institute of Radiological and Medical Sciences; Division for Healthcare Technology Assessment Research (Y-JK, M-SK, SHK, HJT, CMP, AJJ, HJK, JAC, MJK), National Evidence-based Healthcare Collaborating Agency; Department of General Surgery (K-SS), Seoul National University Hospital; Department of General Surgery (SBK); Department of Internal Medicine (CJH, YJK), Korea Institute of Radiological and Medical Sciences; Department of Anatomy (HJC), Seoul National University College of Medicine, Seoul, Korea; and Department of Oncology (MNP), McGill University, Montreal, QC, Canada.

## Abstract

Many preclinical reports and retrospective population studies have shown an anticancer effect of metformin in patients with several types of cancer and comorbid type 2 diabetes mellitus (T2DM). In this work, the anticancer effect of metformin was assessed in hepatocellular carcinoma (HCC) patients with T2DM who underwent curative resection.

A population-based retrospective cohort design was used. Data were obtained from the National Health Insurance Service and Korea Center Cancer Registry in the Republic of Korea, identifying 5494 patients with newly diagnosed HCC who underwent curative resection between 2005 and 2011. Crude and adjusted hazard ratios (HRs) were calculated using Cox proportional hazard models to estimate effects. In the sensitivity analysis, we excluded patients who started metformin or other oral hypoglycemic agents (OHAs) after HCC diagnosis to control for immortal time bias.

From the patient cohort, 751 diabetic patients who were prescribed an OHA were analyzed for HCC-specific mortality and retreatment upon recurrence, comparing 533 patients treated with metformin to 218 patients treated without metformin. In the fully adjusted analyses, metformin users showed a significantly lower risk of HCC-specific mortality (HR 0.38, 95% confidence interval [CI] 0.30–0.49) and retreatment events (HR 0.41, 95% CI 0.33–0.52) compared with metformin nonusers. Risks for HCC-specific mortality were consistently lower among metformin-using groups, excluding patients who started metformin or OHAs after diagnosis.

In this large population-based cohort of patients with comorbid HCC and T2DM, treated with curative hepatic resection, metformin use was associated with improvement of HCC-specific mortality and reduced occurrence of retreatment events.

## INTRODUCTION

Hepatocellular carcinoma (HCC) represents the sixth most common neoplasm and the third leading cause of cancer-related mortality worldwide. Most cases of HCC (80%) arise in eastern Asia and sub-Saharan Africa, where the dominant risk factor is chronic infection with the hepatitis B virus (HBV).^1^ In Korea, HBV is endemic, and although the HCC incidence has declined over the last decade, it remains the fourth most common cancer in Korean men. This results in mortality from liver cancer being the second most common cause of cancer-related death in Korea.^2^

Hepatic resection is the treatment of choice for HCC in individuals without cirrhosis, with 5-year survival rates reported to be ∼50%.^1^ However, expected 5-year intrahepatic recurrence rates have been shown to be up to 70%.^3^ Although survival rates for patients with HCC have improved with advances in surgical techniques and alternative treatments, long-term survival rates remain unsatisfactory due to the high recurrence and metastasis rates.^3^

Type 2 diabetes mellitus (T2DM) increases the risk of developing certain types of cancer, including HCC.^4–6^ Metformin, a biguanide derivative, is one of the most frequently prescribed antihyperglycemic drugs and is used as the first-line therapy for T2DM.^7^ Many retrospective studies have shown an anticancer effect from metformin in several cancer types with T2DM comorbidity.^8,9^ However, the anticancer effect has not been observed in all cancers,^9,10^ and the mechanism of action and beneficial effects of metformin treatment in certain tumor types remains controversial. Therefore, prospective clinical trials are required to determine whether metformin has clinical benefits as an anticancer agent, and trials using adjuvant metformin have been initiated in patients with many cancer types. Both direct and indirect mechanisms of action have been proposed,^11^ and whereas these mechanisms are plausible and suggested by experimental data,^12–14^ the first randomized placebo-controlled clinical trial of metformin carried out for pancreatic cancer showed no benefit.^15^ This result may be related to inadequate drug concentrations in certain tumors, and/or the advanced stage of cancer in patients in this trial. Compared with other cancers, one may anticipate a greater effect of metformin on HCC, given that organic cation transporter 1 (*SLC22A1)* is highly expressed in hepatocytes and can enable increased uptake of metformin in the liver.^16^ However, little is known about the effects of metformin on HCC mortality. To clarify the potential therapeutic effects and decreasing recurrence due to metformin treatment in patients with HCC, a population-based cohort study was conducted, taking advantage of a large-size data set available from the National Health Insurance Service (NHIS) and the Korea Center Cancer Registry (KCCR) in the Republic of Korea. Based on the hypothesis that a low blood concentration of metformin would not be capable of decreasing gross tumor burden,^17,18^ subject selection for the present study was confined to patients who underwent curative hepatic resection, and the effect of metformin on microscopic tumor burden and tumor prevention was evaluated.

## METHODS

### Data Source

Data were initially provided by the KCCR and were linked with national claims data from the NHIS using unique personal identification numbers generated for this study with the consent of the KCCR. The KCCR data covers nationwide cancer cases in the Republic of Korea^19^ and includes the dates and sites of patients’ primary cancer diagnoses. The NHIS has comprehensive data sets for diagnoses, treatments, procedures, surgical history, and prescription records of all insured patients, representing 98% of the entire Korean population. In addition, all those insured and their dependents are required to have periodic general health examinations.

As this study was based on routinely collected administrative data, participant consent was not required. This study was approved by the institutional review board of the National Evidence-Based Healthcare Collaborating Agency.

### Study Population

**FIGURE 1:**
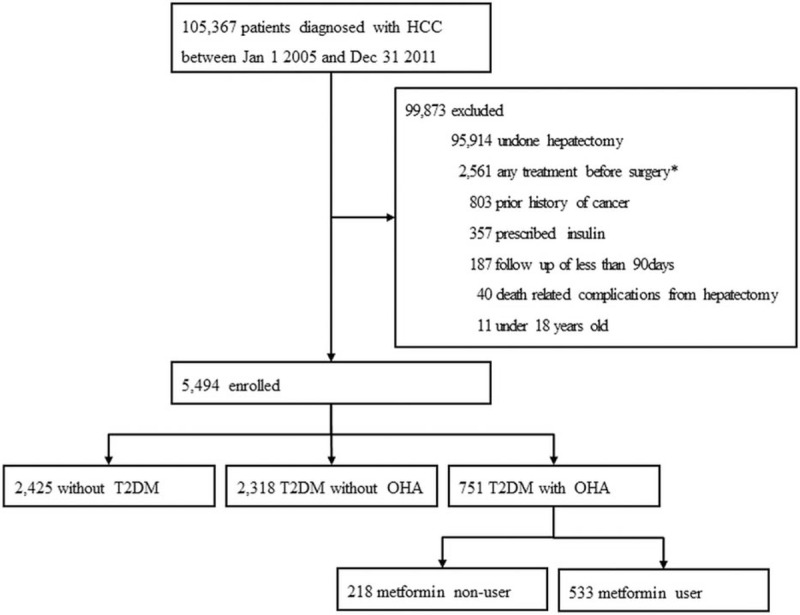
Patient selection flow diagram. ^∗^To identify the exact patients who are at an operable stage upon diagnosis, participants were excluded if they had any treatment before surgery, including TACE, radiofrequency ablation, PEI, radiotherapy, or chemotherapy. HCC = hepatocellular carcinoma, OHA = oral hypoglycemic agent, PEI = percutaneous ethanol injection, RFA = radiofrequency ablation, TACE = transarterial chemoembolization, T2DM = type 2 diabetes mellitus.

The study population initially included 105,367 adults with a primary diagnosis of HCC (International Classification of Diseases, 10th revision [ICD-10], C22) identified from KCCR data between January 1, 2005 and December 31, 2011. Individuals who underwent curative surgical resection for HCC at diagnosis were selected from the NHIS database. First, 95,914 patients who had undergone hepatectomy were excluded. This claim data may have included patients who underwent surgery for tumor recurrence. Therefore, to specifically identify patients who were at an operable stage upon diagnosis, participants were excluded if they received any treatment before surgery, including transarterial chemoembolization (TACE), radiofrequency ablation (RFA), percutaneous ethanol injection (PEI), radiotherapy, or chemotherapy. Patients with a history of invasive cancers other than HCC were also excluded. To exclude patients with more severe diabetes, we excluded insulin users. Patients with a very short follow-up duration, of <90 days were also excluded. Patients who did not survive >30 days after surgery were excluded because of the possibility that they died due to surgical complications. The remaining 5494 patients who underwent curative resection at diagnosis were enrolled for analysis. All included patients were divided into groups with or without T2DM. Patients were included in the T2DM group if they had ≥1 diagnosis of T2DM as noted by an ICD-10 code (E11) in the NHIS data. Patients with T2DM were subdivided into 2 groups based on the use of oral hypoglycemic agents (OHAs). The group of T2DM patients using OHA was further divided into metformin users and metformin nonusers. Details of the inclusion criteria are presented in Figure 1.

### Exposure and Follow-Up

Drug exposure was defined as receiving OHAs in the same class for ≥90 days during the follow-up period. All patients treated with metformin were categorized as “metformin users,” whereas use of other drugs including sulfonylurea, thiazolidinedione, or other OHAs were categorized as “nonmetformin users.” In patients treated with combination therapies, those prescribed metformin for >90 days were categorized as metformin users.

The cohort entry date for each patient was defined as the first date of diagnosis with HCC, and the exit date (censoring date) was the earliest of, the date of death, the date of any clinical event indicating recurrence, 5 years after cohort entry, or end of the study period on December 31, 2013.

### End Points and Definitions

The primary study outcome was HCC-specific mortality. We obtained the data on HCC-specific deaths from the National Population Registry of the Korea National Statistical Office through December 31, 2013, by matching study subjects with the use of unique personal identification numbers. The secondary study outcome was tumor recurrence during follow-up periods. However, we were not able to obtain information regarding follow-up imaging or other medical records. Therefore, we indirectly identified tumor recurrence by analyzing the data for treatments reflecting tumor recurrence and defined these as “retreatment events.” Therefore, we regarded any treatments identified in this manner, which were conducted ≥3 months after the initial hepatic resection as treatment for tumor recurrence. Data for retreatment events were obtained from the NHIS from patients receiving any of the following, hepatectomy, RFA, TACE, PEI, radiotherapy, or chemotherapy.

### Covariates

Information available from the KCCR included diagnosis data providing patient cancer types and dates. All other demographic and clinical information were extracted from claims data or health examination data provided by the NHIS. Cancer-related treatment data, including surgery, RFA, TACE, PEI, radiotherapy, or chemotherapy, and use of diabetes-related medications were obtained from NHIS data. Patients’ body mass index and biochemical values were abstracted from the closest NHIS yearly health examination. Comorbidities occurring from 180 days prior to cancer diagnosis through the date of diagnosis were determined from NHIS claims data. Comorbidities of interest included myocardial infarction, coronary heart disease, peripheral vascular disease, cardiovascular disease, chronic pulmonary disease, rheumatic heart disease, and renal insufficiency/renal failure, which were summarized using a Charlson comorbidity index^20^ indicating the influence of comorbidities other than diabetes and cancer.

### Statistical Analysis

In the main analysis, we compared the baseline characteristics of patients categorized by metformin use. We further compared the rates of HCC-specific mortality and occurrence of retreatment events during the follow-up period among patients with and without metformin using a Cox proportional hazard model and cumulative probability curves derived from Kaplan–Meier estimates. In addition, we divided retreatment events into retreatment <2 years and retreatment ≥2 years after HCC diagnosis. To estimate the rate of retreatment events <2 years, we censored those without retreatment up to 2 years after HCC diagnosis. To examine retreatment events after ≥2 years, we targeted retreatment event-free patients, by excluding those who had retreatment or died before 2 years, and those with a follow-up period of <2 years. We also compared event rates based on the medication possession ratio (MPR), which was defined as total days the target medication was prescribed divided by the total days between prescriptions. An MPR of >80% was considered acceptable adherence. After unadjusted analyses were initially performed, we conducted adjusted analyses, including the potential confounding variables of age, sex, hepatitis B or hepatitis C status, use of antiviral medication, and Charlson comorbidity index.

We conducted several sensitivity analyses, in the first of which we excluded patients who started treatment with metformin or other OHAs after HCC diagnosis to control for immortal time bias.^21^ In the second sensitivity analysis, we evaluated information from health examinations, which were available in 67% of patients, allowing analysis adjusted for the potential confounding variables of age, sex, hepatitis B or hepatitis C status, use of antiviral medication, Charlson comorbidity index, body mass index, total cholesterol, and fasting glucose levels. Furthermore, we performed a restricted analysis among those with hepatitis type B or hepatitis type C to specifically examine the effects in this group at high risk for HCC. In addition, we analyzed sulfonylurea treatment in the same manner as metformin treatment to verify that the effects observed on HCC-specific mortality were specific to metformin and not replicable with any antidiabetic drug. All analyses were performed using Statistical Analysis System software, version 9.3 (SAS Institute, Cary, NC).

## RESULTS

From an initial population of 105,367 HCC patients, 5494 patients received treatment with curative resection and met none of the exclusion criteria. Of those, 751 patients were prescribed OHAs and classified in the T2DM group, with 533 patients receiving metformin for ≥90 days (Figure 1). The median age of the T2DM patients was 60 years, and ∼80% were men. Baseline demographics, including clinical risk factors, comorbidities, or physiological characteristics were comparable between metformin users and metformin nonusers. For HCC patients with T2DM, the total duration of OHA prescriptions was longer for metformin users than for metformin nonusers. In addition, the proportion of patients receiving antiviral medication was higher in the nonmetformin group than in the metformin group (Table 1).

**TABLE 1:**
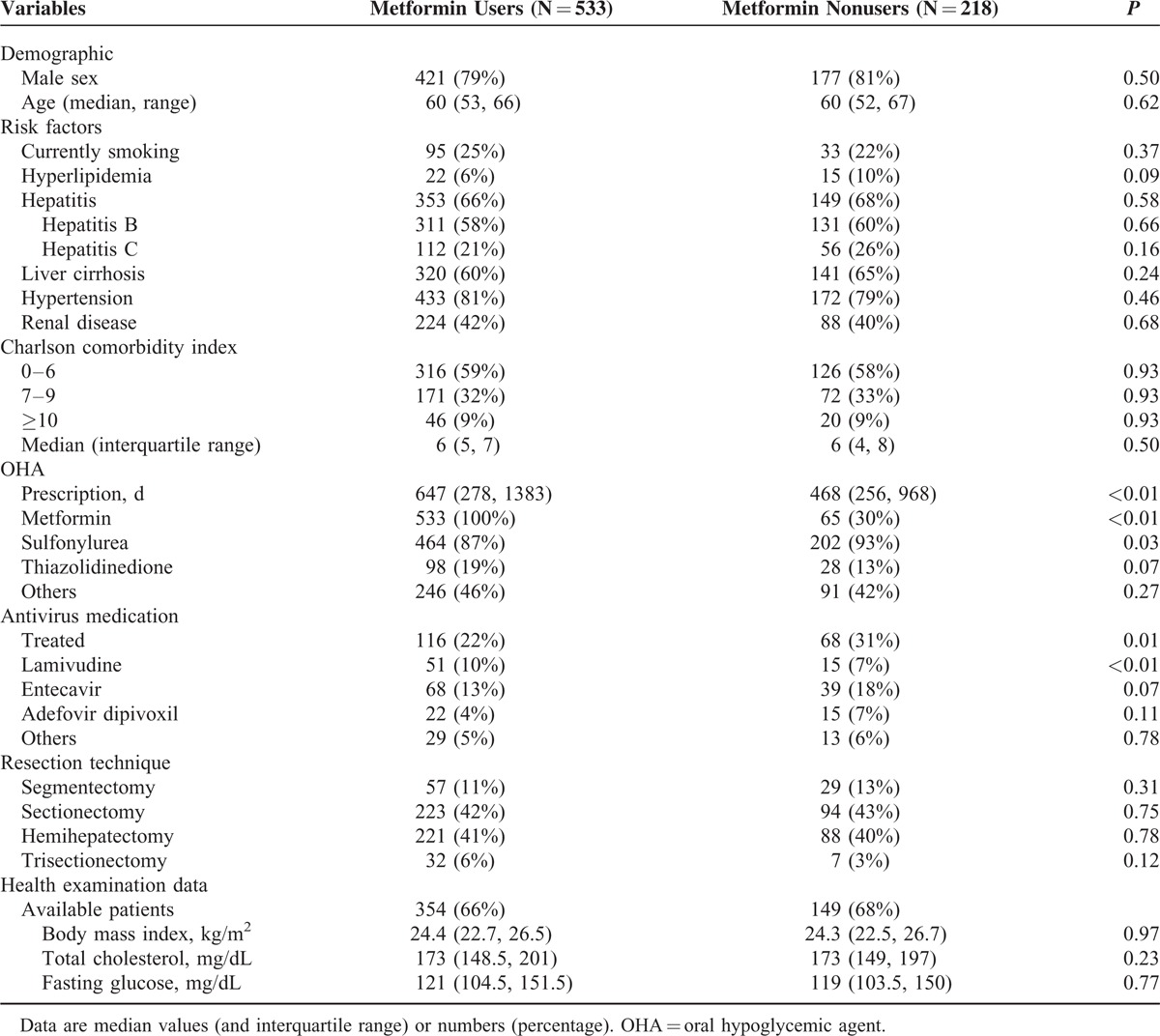
Baseline Characteristics of the Patients by Metformin Use

**FIGURE 2:**
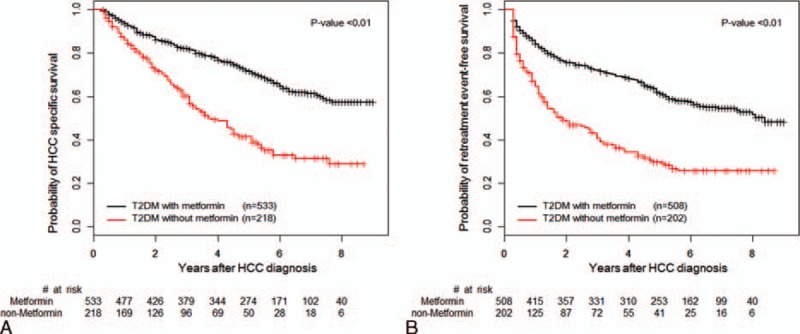
Kaplan–Meier estimation of HCC-specific and retreatment^∗^ event-free survival. HCC-specific (A) and retreatment event-free (B) survival in T2DM who were prescribed OHAs. ^∗^Retreatment event was defined as cancer-related treatment conducted 3 months after initial hepatic resection including surgery, RFA, TACE, PEI, radiotherapy, or chemotherapy. In the retreatment event analysis, patients were excluded whose retreatment event occurs before the initiation of OHA prescription. HCC = hepatocellular carcinoma, OHA = oral hypoglycemic agent, PEI = percutaneous ethanol injection, RFA = radiofrequency ablation, TACE = transarterial chemoembolization, T2DM = type 2 diabetes mellitus.

The HCC-specific survival and retreatment event-free survival was significantly higher among metformin users compared with metformin nonusers in T2DM patients during the follow-up period (Figure 2A and B). In the unadjusted analyses, metformin users showed a significant lower risk of HCC-specific mortality than the metformin nonusers (hazard ratio [HR] 0.40, 95% confidence interval [CI] 0.32–0.51). After multivariable analysis adjusted for clinical covariates, metformin users still had a significantly lower risk of events when compared with metformin nonusers (HR 0.38, 95% CI 0.30–0.49). The adjusted risk for retreatment events was also significantly lower in metformin users (HR 0.41, 95% CI 0.33–0.52) compared with metformin nonusers. Further examination of the relationship between metformin use and the occurrence of retreatment events within two years, or ≥2 years after diagnosis showed that the adjusted risks were also lower for metformin users in both analyses (HR 0.40, 95% CI 0.31–0.53 for recurrence within 2 years; HR 0.52, 95% CI 0.35–0.76 for recurrence after ≥2 years; Table 2).

**TABLE 2:**
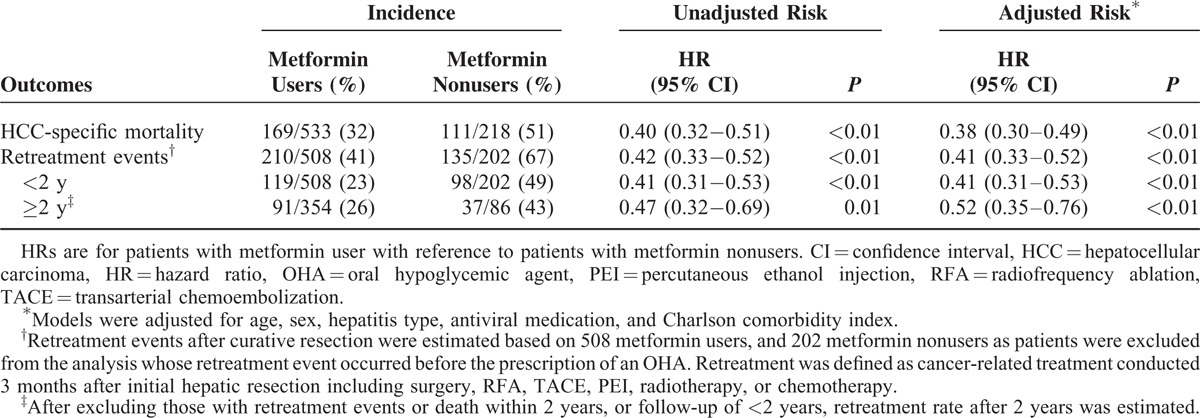
Incidence and HRs of HCC-Specific Mortality and Retreatment Event by Metformin Use

**FIGURE 3:**
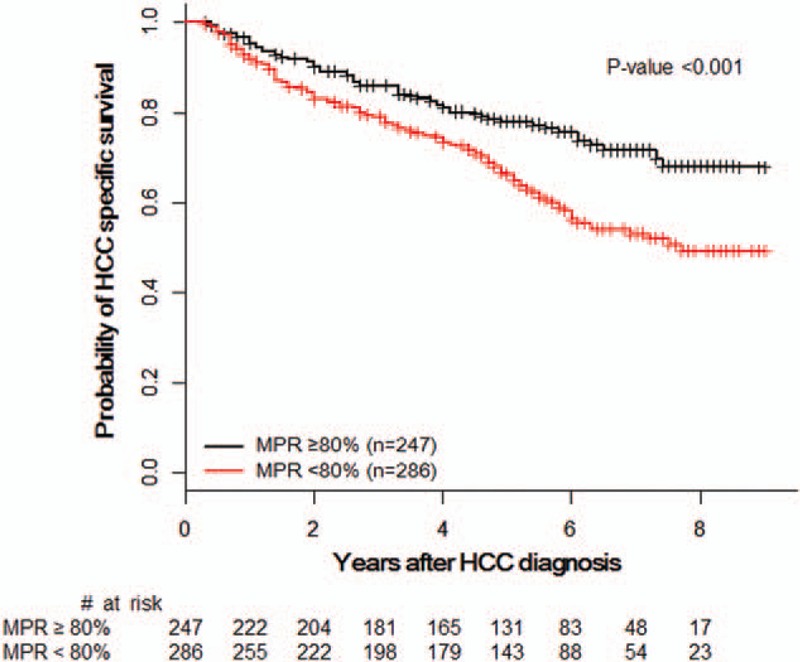
Kaplan–Meier estimation of HCC-specific survival by MPR in metformin user group. HCC = hepatocellular carcinoma, MPR = medication possession ratio.

Subgroup analysis of metformin users found the adjusted risk for HCC-specific mortality to be significantly lower for patients with an MPR of ≥80%, compared with those having an MPR of <80% (HR 0.57, 95% CI 0.41–0.78). A Kaplan–Meier estimation of survival is displayed in Figure 3.

The sensitivity analysis found risks for HCC-specific mortality to be consistently lower among metformin using groups. This held for the grouping that excluded patients who started OHAs after diagnosis, and the group of patients with available health examination data. A subgroup analysis based on hepatitis type showed metformin users had a consistently lower HCC-specific mortality compared with metformin nonusers. Of note, in contrast to metformin, sulfonylurea users did not achieve significantly lower risk for HCC-specific mortality compared with that of sulfonylurea nonusers (Table 3).

**TABLE 3:**
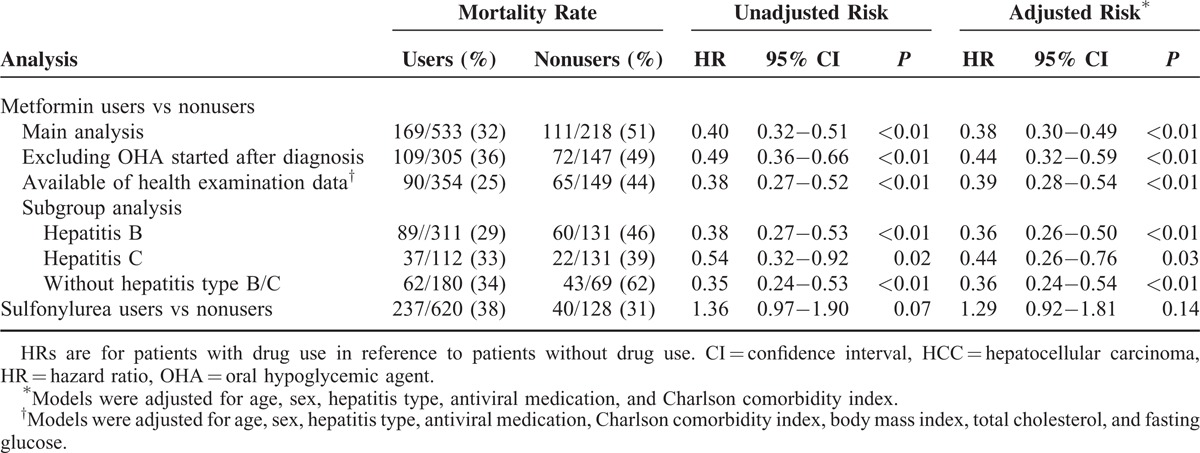
Sensitivity Analyses for Association Between Medication and HCC-Specific Mortality

## DISCUSSION

In this Korean population-based cohort of newly diagnosed diabetic patients with HCC treated with hepatic resection, we observed a significant reduction in the rate of HCC-specific mortality and retreatment events in the T2DM patients who used metformin compared with those who did not use metformin. There have been a limited number of studies examining metformin's effect on survival in HCC.^22,23^ The positive result seen by Chen et al^22^ in HCC patients with T2DM after RFA, where they observed a 76% mortality reduction in metformin users, were criticized for having a small number of patients and lacking controls for immortal time bias.^21^ Bhat et al,^23^ analyzing a relatively large cohort (701 patients) reported that metformin use does not improve survival in HCC. However, that cohort had heterogeneous characteristics with patients at various stages of disease and was missing staging data for some patients. In addition, the authors indicated that their analysis was not adjusted for treatment modalities.

In several epidemiologic studies, metformin use was associated with a reduced incidence of HCC in T2DM patients.^4–6^ A comprehensive meta-analysis of 10 studies reported that metformin use was associated with a 50% reduction in the risk of developing HCC, whereas use of sulfonylureas or insulin did not alter the high risk of developing HCC in patients with T2DM.^24^ Therefore, metformin can be considered a potent inhibitor of de novo tumor development in this carcinogenic environment. Our results confirmed this activity, as metformin use reduced recurrence, measured using retreatment events, occurring >2 years after hepatic resection (Table 2), where recurrence is generally due to de novo tumor formation in the carcinogenic cirrhotic environment.^1,25^ Early phase recurrence, occurring within 2 years of resection, is likely to originate from intrahepatic metastasis of the primary tumor.^1,25^ Our result also showed that metformin reduced retreatment events for these early (<2 years) recurrences (Table 2). These results can be interpreted as metformin having a therapeutic effect on micrometastases and an inhibitory effect preventing the development of de novo tumors. However, the question remains as to whether metformin use in the clinical environment can sufficiently inhibit HCC gross tumor burden.^17,18^

To prevent immortal time bias,^21^ a critical error in pharmacoepidemiological research, we analyzed a metformin user cohort that had been exposed to metformin prior to HCC diagnosis. In the sensitivity analysis, a significant reduction in HCC-specific mortality of metformin users was still observed after excluding patients who started OHAs after diagnosis (Table 3). In addition, the Charlson comorbidity index, use of antiviral medication, and other factors expected to affect prognosis were adjusted in each sensitivity analysis. Considering that hepatitis is endemic to Korea, a subgroup analysis based on hepatitis type was performed; however, the benefit provided by metformin were observed in both types examined (Table 3). In addition, to rule out healthy-user bias, we analyzed outcomes after treatment with sulfonylurea using the same methodology as for metformin treatment^26^ and found sulfonylurea treatment to not be associated with the decrease in HCC-specific mortality (Table 3).

Our study has several strengths. First, the present study has a large sample size, increasing the power of our statistical analysis and minimizing the tendency for selection bias. Second, as treatment strategy of HCC is the strongest prognostic factor influencing outcome,^27^ we therefore only enrolled patients treated with curative hepatic resection and used restrictive inclusion criteria making our patient cohort more homogenous. Third, we showed that patients with a higher MPR (≥80%) for metformin showed a significant reduction in HCC-specific mortality compared with the lower MPR group.

Findings from our study should be interpreted in the context of the following limitations. First, confounding by indication is always a threat to the validity of observational studies. Although we used adjusted models to account for this, we still cannot rule out unmeasured variables such as tumor size, vascular invasion, pathological findings, Child–Pugh score, and tumor markers. Second, dose and exposure duration were not considered, giving the potential for a time-window bias, meaning a patient observed for a longer period is more likely to be exposed to treatment than one observed for a shorter period.^21^ Third, our study did not consider the effect of other drugs that have been reported to relate to cancer mortality, such as aspirin, statins, or bisphosphonates.^28^

In conclusion, in this large population-based cohort of HCC patients with T2DM treated with tumor resection, metformin use was associated with the improvement of HCC-specific mortality and decreased occurrence of retreatment events. To verify the anticancer effect of metformin in patients with HCC, a randomized trial will be necessary in patients without T2DM. The present results serve as background data and rationale for future prospective trials.
